# Biochemical properties of cholesterol aldehyde secosterol and its derivatives

**DOI:** 10.3164/jcbn.17-109

**Published:** 2018-02-07

**Authors:** Noriyuki Miyoshi

**Affiliations:** 1 Laboratory of Biochemistry, Graduate School of Integrated Pharmaceutical and Nutritional Sciences, University of Shizuoka, Shizuoka 422-8526, Japan

**Keywords:** secosterol, protein adduct, LC-MS, inflammation, biomarker

## Abstract

Elevated levels of cholesterol aldehyde, 3β-hydroxy-5-oxo-5,6-secocholestan-6-al (secosterol-A, also called 5,6-secosterol), and its aldolization product (secosterol-B) have been detected in human atherosclerotic plaques and tissues samples of brains affected by neurodegeneration, such as Alzheimer’s disease and Lewy body dementia suggesting that increased formation of these compounds may be associated with inflammation-related diseases. Secosterol-A and secosterol-B, and also further oxidized products seco-A-COOH and seco-B-COOH induce several pro-inflammatory activities *in vitro*. Accumulating evidences demonstrate that the covalent bindings of these secosterols to target proteins seem to be critical to trigger their pro-inflammatory activities. One of the molecular mechanisms of protein adduct formations is that aldehydic function of secosterol-A and secosterol-B is reactive and form Schiff bases with ε- or N-terminal amino groups of proteins. In other cases, it is recently suggested that Michael acceptor moiety formed by the dehydration of not only secosterol-A and secosterol-B but also seco-A-COOH may react with nucleophilic site on target proteins. In this review, I summarize and provide an overview of formation mechanism of secosterols in *in vitro* and *in vivo*, patho- or physiological concentrations in biological and clinical samples, and molecular mechanisms of pro-inflammatory activities of secosterols.

## Introduction

Oxysterols are derivatives of cholesterol containing one or more oxygen atoms, other than the OH group on C_3_, as hydroxyl, keto, epoxide or peroxide group—that is mounted on the A and B ring or on the side chain. Oxysterols can be generated either enzymatically, mainly by the group of cytochrome (CYP) P450 family, or by autoxidation.^(1)^ In brief, in biological systems oxygenation on side-chain is almost exclusively enzymatic, while that on the A and B ring can occur both enzymatically and by autoxidation.

**Fig. 1:**
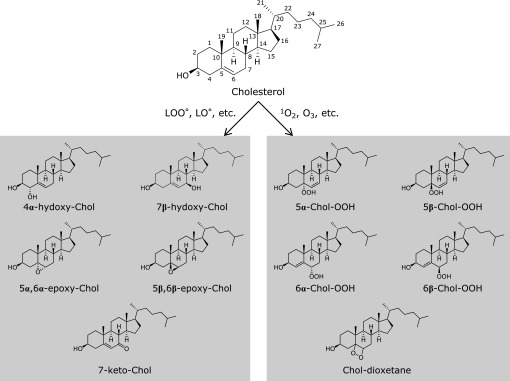
Chemical structure of cholesterol autooxidation products.

Oxysterols arising from enzymatic synthesis can be used as markers of their respective cytochrome activity. On the other hand, the susceptibility of cholesterol to non-enzymatic oxidation has generated considerable interest in oxysterols as potential markers for the non-invasive study of oxidative stress *in vivo*. Additional interest in oxysterols stems from the biological activity of many oxysterols that is useful to elucidate pathophysiological pathways in human diseases and for pharmacological application.^(2)^ Cholesterol autoxidation proceeds via two distinct pathways, a free radical pathway driven by a chain reaction mechanism (type I) and a non-free radical pathway (type II), which is driven stoichiometrically by reactive oxygen species (Fig. 1).^(2,3)^

Type I autoxidation involves initiation and propagation reactions. Free radicals provide the initiation step by hydrogen abstraction, formation of a carbon centered radical and subsequent oxygen capture. Afterwards, the process advances through free radical intermediates—including, peroxyl radicals (LOO^•^) and alkoxyl radicals (LO^•^)—that in turn recruit additional non-oxidized molecules and provoke the spreading of the process via a chain-reaction, the propagation phase. A multitude of oxysterols can be formed upon type I autoxidation but analytical issues restrain the number of species usable as markers of oxidative stress in biological matrices. The species that actually perform well on GC/MS, which is the gold standard for oxysterols measurement, are: 4α- and 7β-hydoxycholesterol, 5α,6α- and 5β,6β-epoxides, and 7-ketocholesterol.^(2)^ Recent studies from Porter and co-workers have established the product distribution of several oxysterols obtained through the free radical chain oxidation of the cholesterol precursor 7-dehydrocholesterol.^(4)^

In type II autoxidation the main molecules that are involved in cholesterol oxidation are the non-radical species singlet oxygen and ozone. Singlet oxygen is formed by an input of energy, such as photoactivation, the Russell mechanism, based on the decomposition of lipid hydroperoxides, and by the reactions of hypochlorous acid (HOCl) and hydrogen peroxide. Four primary species are possible in the reaction of cholesterol with singlet oxygen via ene addition: 5α-cholesterol-hydroperoxide (5α-Chol-OOH), 5β-cholesterol-hydroperoxide (5β-Chol-OOH), 6α-cholesterol-hydroperoxide (6α-Chol-OOH), and 6β-cholesterol-hydroperoxide (6β-Chol-OOH), and Chol-dioxetane. The formation of 5α-Chol-OOH is highly favored at a rate of approximately one order of magnitude higher than that of 6α-Chol-OOH and 6β-Chol-OOH.^(5)^ Minor products of ozone-driven cholesterol oxidation, are 5α,6α- and 5β,6β-epoxides, have been found to form in ethyl acetate,^(6)^ but their participation in physiological environment is not reported. The 7α- and 7β-Chol-OOH are formed during the reaction of singlet oxygen with cholesterol and generated indirectly by the allylic rearrangement of 5α-Chol-OOH,^(7)^ which takes place at high peroxidation levels but is negligible under limited cholesterol oxidation (<5%).^(8)^ Cholesterol hydroperoxides are susceptible to 1 e^−^ reduction that gives rise to alkoxyl- and peroxyl-radical intermediates that, in turn, can trigger chain reactions and amplify the free radical cascade of cholesterol oxidation. All cholesterol hydroperoxides are expected to be equally susceptible to 1 e^−^ reduction in the presence of metal catalysts. Similar rate constants have been reported for the reduction of 5α-Chol-OOH and 6α-Chol-OOH formation during incubation with an iron-based redox cycling system in a homogeneous solution in which cholesterol was the only chain-carrying species.^(5)^ The potency of 5α-Chol-OOH and 7α-Chol-OOH as chain initiators is comparable.^(9)^ Cholesterol hydroperoxides (Chol-OOHs) are resistant to direct 2 e^−^ reduction that is catalyzed by Se-dependent glutathione peroxidase.^(10)^ This means that Chol-OOHs have a potential long half-life in cells. The only enzyme capable of catalyzing the reduction of Chol-OOHs to stable diols, is the phospholipid-hydroperoxide glutathione peroxidase (PHGPx).^(11)^ However, the reduction of Chol-OOH by PHGPx is 6 times slower compared to the reduction of phospholipid hydroperoxides,^(12)^ and shows different rate constants ranging from 0.8 × 10^2^ min^−1^ for 5α-Chol-OOH to ≈ 6 × 10^2^ min^−1^ for 6α-Chol-OOH and 6β-Chol-OOH.^(5)^ Thus, 5α-Chol-OOH results the most abundant product of singlet oxygen reaction with cholesterol, and the least resistant to detoxification via PHGPx. The forward products arising from type-II cholesterol autoxidation are cholesterol aldehydes, called secosterol described in detail below.

## Proposed Mechanisms for the Formation of Secosterol-A and -B

**Fig. 2:**
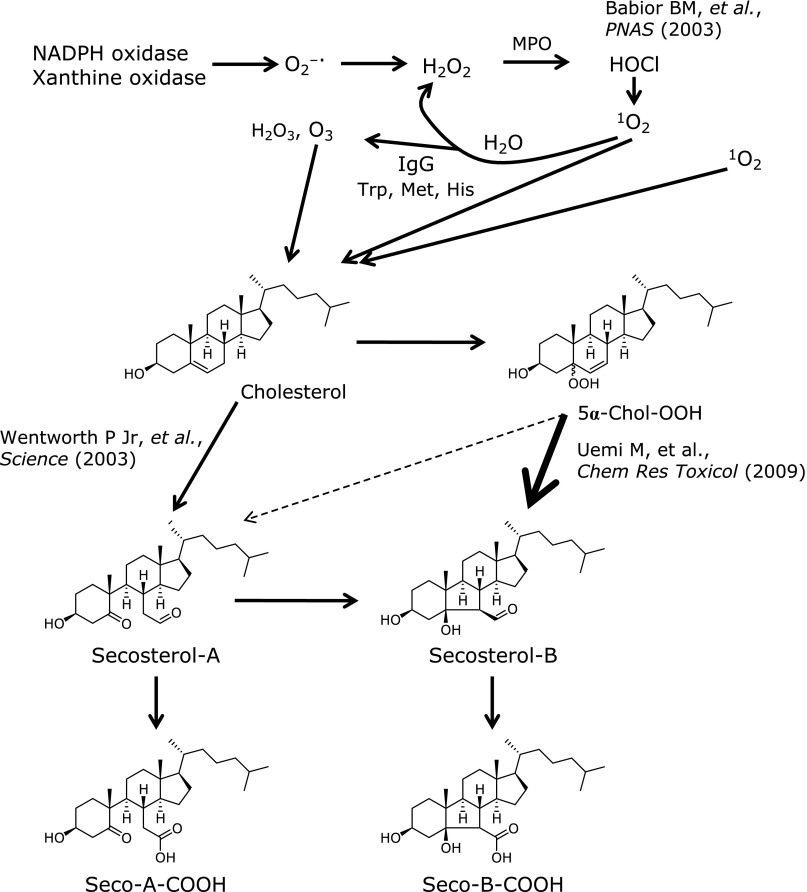
Chemical structure and formation pathway for secosetrol-A and secosterol-B and their derivatives. Major pathway to form secosterol-A is oxidation of cholesterol with ozone produced by reactive oxygen species cascade. On the other hand, main product in the reaction of cholesterol and singlet oxygen is 5α-Chol-OOH, most of which is therefore converted to secosterol-B. Although some part of secosterol-A is converted to secosterol-B, other part of secosterol-A and secosterol-B are oxidized to form seco-A-COOH and seco-B-COOH, respectively.

3β-Hydroxy-5-oxo-5,6-secocholestan-6-al (secosterol-A) and its aldolization product 3β-hydroxy-5β-hydroxy-B-norcholestane-6β-carboxaldehyde (secosterol-B) is the major cholesterol ozonolysis products (Fig. 2).^(6)^ Wentworth *et al.*^(13)^ conducted pioneering works on secosterol-A and secosterol-B as potential diagnostic markers of endogenous ozone production. They proposed a mechanisms for the formation of ozone *in vivo* consisting of reactive oxygen species cascade: (a) superoxide generation by activated neutrophil, (b) dismutation into hydrogen peroxide, (c) HOCl formation by myeloperoxidase (MPO), (d) singlet oxygen generation by the reaction of HOCl and hydrogen peroxide, and afterwards (e) formation of ozone from the singlet oxygen in an antibody-catalyzed water oxidation pathway (Fig. 2).^(14,15)^ A similar mechanism for the production of ozone-like reactive species from singlet oxygen in an amino acids-catalyzed water oxidation pathway was also reported.^(16)^ However others have argued against the ozone-dependent mechanism of secosterol-A formation *in vivo*,^(17–19)^ and pointed out an alternative pathway for the formation of secosterol-A and secosterol-B. Uemi *et al.*^(20)^ proposed a mechanism based on the Hock cleavage of 5α-Chol-OOH or a Chol-1,2-dioxetane intermediate formed by the reaction of cholesterol with singlet oxygen. Our previous work provided evidence for secosterol-A and secosterol-B generation by the reaction of cholesterol with singlet oxygen produced by 1-methylnaphthalene-4-endoperoxide (MNPE) in phosphate buffer, and by the MPO-H_2_O_2_-Cl^−^ system.^(21)^ Taken together these data point to a duplicate mechanism of secosterol-A and secosterol-B formation involving either ozone or singlet oxygen. Being secosterol-B the predominant species formed during singlet oxygen-mediated cholesterol oxidation (secosterol-B is about 5–10 times higher than secosterol-A)—the ratio secosterol-A to secosterol-B has been proposed as a surrogate measure to decipher the ozone-dependent and independent oxidation of cholesterol.^(21)^ On the other hand, secosterol-A occurs as the dominant species formed by ozone in aqueous buffer system,^(22)^ and by PMA-activated neutrophil in culture.^(23)^ In addition, we were able to observe a time-dependent elevation of secosterol-A and secosterol-B in plasma after injecting lipopolysaccharide to C57BL/6J mice, but not in MPO-deficient mice. Besides, basal levels of secosterol-A and secosterol-B in the plasma of MPO-deficient mice were lower than the value found in wild-type mice, but secosterol-A was barely detectable.^(23)^ Secosterol-B was shown to be formed by aldolization of secosterol-A and, also, in an ozone-independent pathway via 5α-OOH-Chol or Chol-dioxetane.^(20)^ Secosterol-B detected in the plasma of MPO-deficient mice, therefore, could be formed by the reaction of cholesterol with singlet oxygen generated *in vivo*, although how and where the oxidant from is currently unknown. Taken together these findings advise the occurrence of ozone-mediated reaction *in vivo* but no conclusive evidences so far could be drawn for ozone production *in vivo*.

## Derivatives of Secosterols

Secosterol-A is unstable in physiological aqueous conditions, such as culture medium containing serum, and is readily converted to its aldolization product secosterol-B. In part, secosterol-A and secosterol-B are further converted to their oxidized forms 3β-hydroxy-5-oxo-secocholestan-6-oic acid (seco-A-COOH) and 3β-hydroxy-5β-hydroxy-B-norcholestane-6-oic acid (seco-B-COOH) in culture media and probably *in vivo*.^(23)^ Additionally, Windsor and co-workers recently proposed that secosterol-A and secosterol-B could be received dehydration before their protein adducts formation. The detailed mechanisms of protein adduct by the dehydrated secosterols are discussed in detail below. Moreover, ozonolysis products of the major cholesteryl fatty acid esters transported in human LDL have been reported.^(24)^ Under a flux of ozone, cholesteryl palmitate gives rise to palmitoyl-secosterol-A and palmitoyl-secosterol-B. Instead, ozonolysis of cholesterol esterified with unsaturated fatty acids oleate and linoleate admits the initial isolation of cholesteryl-9-oxononanoate and the subsequent appearance of both the fatty acid and cholesteryl moiety oxidation products, i.e., 9-oxononanoyl-secosterol-A and 9-oxononanoyl-secosterol-B.^(24)^ These compounds derived from cholesterol/cholesterol esters ozonolysis exert potent biological activities including the denaturation of proteins and strong cytotoxicity in different cells lines (see below).

## Endogenous Levels of Secosterol-A and -B

High levels of secosterol-A and secosterol-B have been detected in human atherosclerotic plaques^(13)^ and tissues samples of brains affected by neurodegeneration, such as Alzheimer’s disease and Lewy body dementia,^(25,26)^ suggesting that increased formation of these compounds may be associated with inflammation-related diseases.^(27,28)^ For the analysis of secosterol-A and secosterol-B in clinical samples, HPLC separation with UV, fluorescence, or MS(/MS) detection have been widely employed. In general, lipid extracts of blood or tissues containing secosterol-A and secosterol-B are derivatized with hydrazine derivatives, such as dinitrophenylhydrazine (DNPH), then injected to HPLC or LC-MS.^(13,29)^ For the higher sensitive detection, derivatization with dansyl (DNSL) hydrazine (LOD = 1 fmol),^(21,23,26)^ 1-pyrenebutyric hydrazine (PBH; LOD = 10 fmol),^(30)^ Girard P (GP) hydrazine (LOD = 2.7 fmol),^(31)^ or 2-hydrazino-1-methylpyridine (HMP; LOD = 10–50 amol)^(32)^ are applicable. Using these derivatizing reagents, secosterol-A and secosterol-B present in blood or tissues were detectable as secosterol-hydrazone derivatives by HPLC-fluorescence detector and LC-MS/MS. For example, Griffiths and co-workers reported levels of seco-A and seco-B in rat brain of ~100 pg/mg (240 pmol/g) and ~300 pg/mg (720 pmol/g), respectively, determined after derivatization with GP hydrazine.^(31)^ Secosterol-A and secosterol-B in human brain were also analyzed by HPLC-UV or LC-MS after derivatization with DNPH resulting in levels of (secosterol-A + secosterol-B) 0.44 pmol/mg in Alzheimer’s patients (*n* = 4) and 0.35 pmol/mg in control subjects (*n* = 7).^(25)^ In addition, increased levels of secosterols (secosterol-A + secosterol-B) were observed in the cortex of brain affected by Lewy body dementia (0.213 µM, *n* = 15) compared to those of age-matched controls (0.093 µM, *n* = 18) in analysis done by HPLC-fluorescence detector and LC-MS after DNSL derivatization.^(26)^ Wentworth and co-worker analyzed DNPH-derivatives of secosterol-A and secosterol-B in organic extracts of human atherosclerotic plaque by HPLC coupled with MS, and found them in the ranges of 6.8–61.3 pmol/mg plaque.^(13)^ Elevated levels of secosterol-B were also observed in the plasma of these patients (70–1,690 nM) compared to those of controls subjects.^(13)^ We have recently developed a highly sensitive isotope dilution method to detect secosterol-A and secosterol-B as HMP derivatives by LC-ESI-MS/MS, and using 3,4-^13^C-seco-A and 3,4-^13^C-seco-B as internal standards.^(32)^ We found levels of secosterol-A and secosterol-B of 23.6 ± 16.6 nM and 27.3 ± 41.0 nM, respectively, in human plasma (*n* = 10). The levels of secosterol-A and secosterol-B were respectively 1.4 ± 0.7 nM and 4.3 ± 0.8 nM in the plasma, 10.4 ± 16.3 pmol/g and 110.9 ± 10.6 pmol/g in the brain, 34.1 ± 21.6 pmol/g and 161.5 ± 56.3 pmol/g in the liver and 29.1 ± 1.3 pmol/g, and 80.4 ± 1.4 pmol/g in the lung of C57BL/6J mice (*n* = 3). The higher levels of secosterol-A and secosterol-B in human plasma compare to that of mouse could be due to the environmental factor such as sun light exposure and some pathogens, which are potential triggers to generate endogenous reactive oxygen species. In addition, ozonolysis products of cholesteryl-oleate and cholesteryl-linoleate, 9-oxononanoyl-secosterol-A and 9-oxononanoyl-secosterol-B, were found in human LDL at levels of 16.5 ± 5.4 and 11.3 ± 3.9 pmol/mg LDL protein, respectively.^(24)^ Notably, the quantitative values of secosterol-A and secosterol-B in biological samples differ widely among the different laboratories. As secosterol-A is very unstable, at least the use of stable isotope labeled internal standards in secosterol analysis is mandatory.

## Chemical and Biological Properties

It is assumed that the aldehydic function of secosterols is reactive and form Schiff bases with ε- or N-terminal amino groups of proteins and with phosphatidylethanolamine. Wentworth *et al.*^(13)^ reported that incubation of human LDL with either secosterol-A or secosterol-B led to time-dependent changes in the circular dichroism spectra of apoB-100, consistent with an altered secondary structure, and increased atherogenicity, e.g., the secosterol-modified LDL was avidly taken up by macrophage leading to foam cell formation. Secosterol-A was shown to randomly modify the 6 different Lys residues of ApoC-II, as well as apolipoprotein that in the absence of lipids has conformational instability and undergoes fibrillization.^(33)^ Secosterol-A accelerated ApoC-II polymerization with concurrent increase in thioflavin fluorescence,^(33)^ a signature of amyloidogenesis.^(34)^ Interestingly, seco-A-COOH, which lacks the aldehyde group and is unable to form Schiff bases, was also able to accelerate ApoC-II fibril formation, albeit at a lesser extent, suggesting that non-covalent mechanisms may support secosterol-dependent ApoC-II amyloidogenesis.^(33)^ These findings are relevant to the mechanisms of atherosclerosis because amyloid deposits are present in 50–60% of atherosclerotic lesions^(35)^ and ApoC-II is a prominent component of these deposits.^(36)^ Concentrations of secosterols are reportedly elevated in the cortex of patients with Lewy body dementia,^(26)^ a disease associated with intra-neuronal accumulation of α-synuclein in the form of amyloid fibrils or Lewy bodies. Secosterol-A, secosterol-B, and seco-A-COOH have been shown to accelerate α-synuclein aggregation *in vitro*, and more interestingly seco-A-COOH, which lacks the aldehyde functionality, was even more potent in forwarding the process.^(26)^ Amyloidogenicity of amyloid-β (Aβ) is considered a crucial player of Alzheimer disease but an open question is the 2–3 order of magnitude disparity between the critical concentration to induce aggregation, which is in the micromolar range, and the actual concentration of Aβ at tissue level, which is in the nanomolar range.^(37)^ Secosterols have been shown to effectively reduce this critical concentration of Aβ for aggregation below 100 nM.^(25,38)^ Lys-16 Aβ modified with secosterols formed amorphous aggregates fastest and at very low concentrations of Aβ (20 nM), followed by the Lys-28 and Asp-1 conjugates. Besides, the aggregates resulting from Aβ Lys-cholesterol aldehyde adducts were more toxic to primary rat cortical neuron.^(39)^ Secosterol-A and secosterol-B in brain samples of patients affected by neurodegenerative disease approach concentrations of up 1 µM^(25,31)^ that are suitable to covalently modify Aβ and increase its amyloidogenicity.^(25,26,38,40,41)^ Secosterol-A and secosterol-B have been reported to induce structural change to myelin basic protein (MBP) relevant to the context of demyelinating diseases.^(42)^ MBP accounts for approximately 30% of the total myelin protein, and is responsible for adhesion and stabilization of the intracellular surfaces of myelin layers. By reacting with MBP, secosterols have been shown to increase the surface exposure of the immunodominant epitope, decrease the surface exposure of the cathepsin D binding, and reduce the size and structural stability of MBP-induced aggregates. As a consequence of these alterations in the structure and function, MBP becomes unable to maintain the integrity of the myelin sheath and vulnerable to autoimmune attack. In line with what observed with secosterol-initiated misfloding of Aβ and α-synuclein, secosterol-A and secosterol-B have been reported to induce misfolding of wild-type *p53*.^(43)^ The tumor suppressor protein *p53* functions to maintain the integrity of the genome, and its activation in response to DNA damage promotes cell-cycle arrest in G1 phase or apoptosis. Upon incubation with secosterols, *p53* undergoes polymerization and forms amyloid fibrillar aggregates. This misfolding renders *p53* unable to bind to DNA and to induce transactivation of *p21*.^(43)^ Given that inflammation is the fuel for secosterols formation and inflammation functions in all stages of tumor development, cholesterol aldehydes provide a chemical link to understand cancer carrying inactive *p53*.

Light-chain deposition disease is a severe, often fatal, clinical condition in which amyloid or amorphous deposits, as a consequence of antibody light chain aggregation, accumulate in the heart and/or kidney.^(44–46)^ Secosterol-A and secosterol-B have been reported to accelerate aggregation of human antibody kappa and lambda light chains *in vitro* under physiologically relevant conditions, causing an amorphous-type aggregation that is thioflavin and Congo red negative for both the kappa and lambda light chains.^(47)^ Given the inflammatory microenvironment of secosterol production and its association with antibodies, the secosterol-induced protein misfolding is consistent with pathophysiological role in light-chain deposition disease.

While the above reported studies show secosterols as playing deleterious roles by promoting misfolding of varied proteins, secosterol-B has unexpectedly been shown to inhibit the misfolding of a truncated murine mutant prion protein. Incubation of secosterol-B with a murine prion protein, paradoxically, induced stabilization of the native form of the prion and inhibited the generation of the disease-causing scrapie form.^(48)^ The inhibition was specific for secosterol-B, where structural analogues were ineffective, offering a promising tool to develop new pharmacological active compounds to treat prion disease.

Additionally, secosterols have been reported to affect membrane and enzyme function. It was shown that secosterols bound phosphatidylethanolamine and phosphatidylserine via Schiff base formation, and also reduced biophysical parameters of membrane stability, which could be associated with various pathogenic insults.^(49–51)^ Secosterol-A, but not secosterol-B, reportedly inhibited endothelial- and neuronal-type NOS activities, probably mediated by adduct formations with lysine residues on these enzymes.^(52)^

**Fig. 3:**
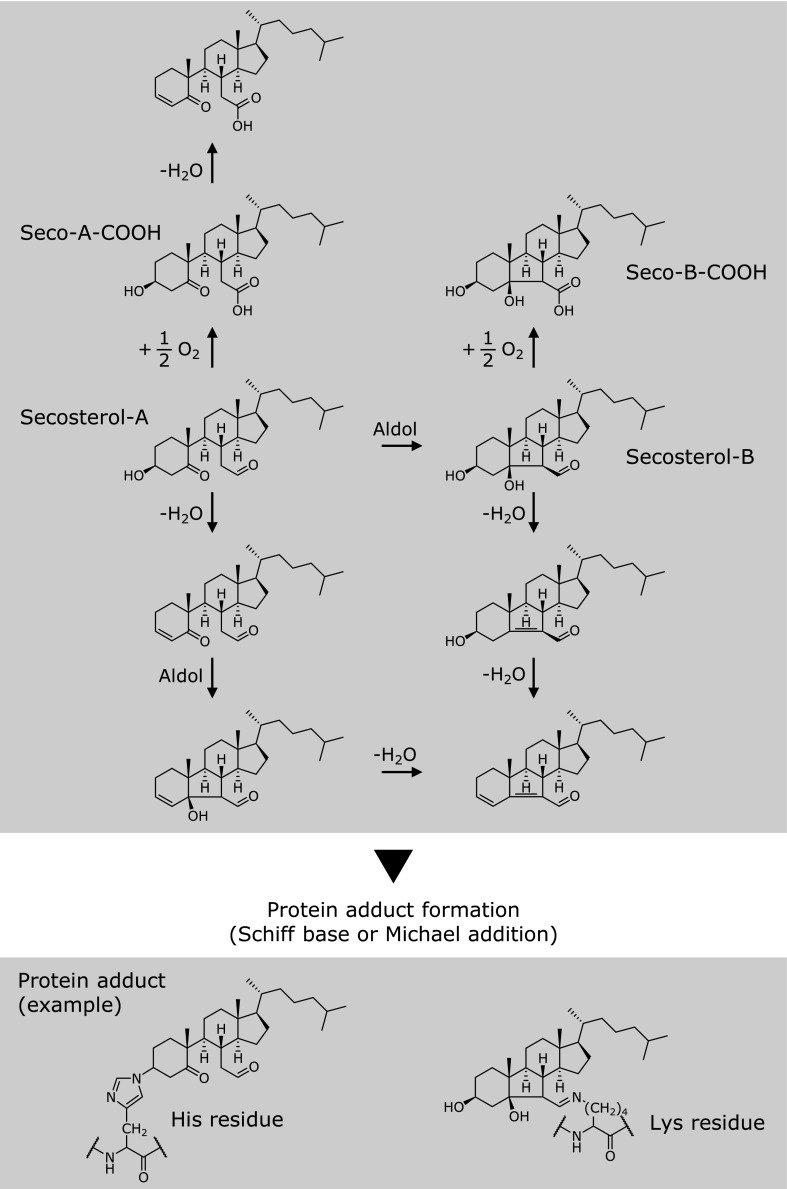
Schematic representation of secosterols oxidation and dehydration and their protein adducts.

Previously Genaro-Mattos and co-workers reported that secosterol-B covalently bound to Lys-22 on cytochrome *c* (cyt *c*) under SDS micellar conditions, which role could be an anchor to mitochondrial membranes.^(53)^ Further study was recently reported from the same group, they performed excellent study about protein adducts of secosterol-A and secosterol-B.^(54)^ They found His residue (His-33) on cyt *c* is one of other targets residue for secosterols, which is the first report to show that secosterol enable to modify a nonlysine residue. His-33 on cyt *c* is known to be a modification site for lipid electrophile, for example 4-hydroxynonenal (HNE), although, unlike HNE, neither secosterol-A nor secosterol-B contains a Michael acceptor, a typical site of nucleophilic attack by histidine. They carefully examined the molecular mechanism of this His adducts, in which they finally found that dehydrated secosterol-A or secosterol-B showed electrophilicity, and are promising active compound to form protein adduct via covalent binding to His residue (Fig. 3). Additionally they demonstrated that LC-MS/MS analysis of covalently modified seco-adducted peptide showed a unique fragmentation pattern of neutral loss 390 Da. This characteristic neutral loss pattern may be a useful criterion to identify seco-adducted protein in proteomic analysis.

Another approach to identify the secosterol-protein adduct was also reported. Speen and co-worker prepared alkynyl-tagged secosterols to discriminate secosterol-protein adduct by click chemistry and found that secosterol-A adducted to liver X receptor (LXR).^(55)^ This adducts formation inactivates LXR activities, which could be associated with secosterol-induced proinflammatory activities.

The biochemical and biophysical properties of secosterols could be associated with their noxious activity on cells. Several studies have found that secosterol-A and secosterol-B induce cell death in various cell lines, including human B-lymphocytes (WI-L2), T-lymphocytes (Jurkat), vascular smooth muscle cells (VSMC), abdominal aorta endothelial cells (HAEC), murine tissue macrophages (J774.1), and an alveolar macrophage cell line (MH-S).^(13)^ Sathishkumar *et al.*^(56)^ reported that secosterol-A exerted about 2-fold higher cytotoxicity than 5,6β-epoxycholesterol in hypothalamic neuron GT1-7 cells. Several pathways have been postulated for secosterol-triggered cell death, including the caspase-3/7-dependent pathway and the mitochondrial and death receptor pathway in cardiomyocyte H9c2 cells,^(57,58)^ the reactive oxygen species-dependent pathway in hypothalamic neuron GT1-7 cells,^(56,59)^ a mitochondrial death pathway in macrophage J774 cells, and the mitogen-activated protein kinase pathway in hepatocarcinoma HepG2 and Huh7 cells.^(60)^ Moreover, seco-A-COOH and seco-B-COOH showed strong cytotoxic activities in human acute promyelocytic leukemia HL-60 cells.^(61)^ Recently, it has been reported that 9-oxononanoyl-secosterol-A and 9-oxononanoyl-secosterol-B—ozonolysis products of cholesteryl-oleate and cholesteryl-linoleate present in human LDL—exert potent cytotoxicity towards HL-60 cells.^(24)^ Their activity is stronger than other cytotoxic oxysterols exhibiting EC50s of 10–20 µM, which were very similar against various cell lines tested.

## Conclusion

Although formation mechanisms of secosterols are not still fully unveiled, elevated levels of secosterols have been observed in various tissues collected from different inflammatory diseases. Secosterol-A, secosterol-B, and other related compounds including seco-A-COOH, seco-B-COOH, and 9-oxononanoyl secosterols exert strong biological activities compared to other oxysterols, such as 5,6-epoxycholesterol or 7-ketocholesterol. Several proteins or peptides including apoB-100, ApoC-II, Aβ, α-synuclein, and etc were denatured by the interaction with secosterols. Because secosterol-A and secosterol-B possess aldehyde group, they enable to covalently bind to Lys residue on target protein to form secosterol-protein adducts. Then, proteins modified by secosterols are insolubilized in aqueous solution by hydrophobicity of secosterols. However it is observed that non-aldehydic seco-A-COOH was also able to accelerate ApoC-II fibril formation and α-synuclein aggregation. This could be due to the α,β-unsaturated carbonyl formation on seco-A-COOH after dehydration (Fig. 3). As reported by Windsor *et al.*^(54)^, seco-A-COOH as well as secosterol-A and secosterol-B but not seco-B-COOH are probably able to form active carbonyl group, then this Michael acceptor may react with nucleophilic target. Therefore, although further study is required, the molecular mechanism and manner of protein adduct formation would be depending on the presence of aldehyde group and dehydration of C3 position in secosterol structure.

## Abbreviations

Aβ: amyloid-β
5α-Chol-OOH: 5α-cholesterol-hydroperoxide
6α-Chol-OOH: 6α-cholesterol-hydroperoxide
5β-Chol-OOH: 5β-cholesterol-hydroperoxide
6β-Chol-OOH: 6β-cholesterol-hydroperoxide
CYP: cytochrome
DNPH: dinitrophenylhydrazine
DNSL: dansyl
GP: Girard P
HMP: 2-hydrazino-1-methylpyridine
HNE: 4-hydroxynonenal
HOCl: hypochlorous acid
MBP: myelin basic protein
MNPE: 1-methylnaphthalene-4-endoperoxide
MPO: myeloperoxidase
PHGP: phospholipid-hydroperoxide glutathione peroxidase
seco-A-COOH: 3β-hydroxy-5-oxo-secocholestan-6-oic acid
seco-B-COOH: 3β-hydroxy-5β-hydroxy-B-norcholestane-6-oic acid
secosterol-A: 3β-hydroxy-5-oxo-5,6-secocholestan-6-al
secosterol-B: 3β-hydroxy-5β-hydroxy-B-norcholestane-6β-carboxaldehyde
